# The complete mitochondrial genome sequence and phylogenetic analysis of *Luciola lateralis*, one of the most famous firefly in Japan (Coleoptera: Lampyridae)

**DOI:** 10.1080/23802359.2017.1365640

**Published:** 2017-08-17

**Authors:** Juri Maeda, Dai-Ichiro Kato, Kazunari Arima, Yuji Ito, Atsushi Toyoda, Hideki Noguchi

**Affiliations:** a Department of Chemistry and Biosciences, Graduate School of Science and Engineering, Kagoshima University, Kagoshima, Japan;; b Advanced Genomics Center, National Institute of Genetics, Mishima, Japan;; c Center for Genome Informatics, Joint Support-Center for Data Science Research, Research Organization of Information and Systems, Mishima, Japan

**Keywords:** *Luciola lateralis*, firefly, Lampyridae, mitochondrial genome, Coleoptera

## Abstract

We will report the complete mitochondrial genome sequence of Japanese firefly ‘Heike Botaru’, *Luciola lateralis* (Coleoptera: Lampyridae). Total length of this mitogenome was 16,719 bp and the composition of each base was A (42.50%), C (9.01%), G (14.16%), T (34.33%), respectively. The obtained sequence fulfils general mitogenome composition of metazoan (13 protein coding sequences (CDSs), 22 tRNA genes, two rRNA subunits, and an AT-rich region). From the phylogenetic tree analysis using 25 kinds of insect mitogenome including firefly family was found that *L. lateralis* is the closest to the genus *Aquatica*.

*Luciola lateralis*, which is called ‘Heike Botaru’ in Japanese, is one of the most famous firefly in Japan. This firefly is classified as an aquatic firefly and its habitat area is not only Japan, but extends to East Siberia and South Korea (Suzuki et al. 2004). In this paper, we will report the complete mitochondrial genome sequence of *L. lateralis* and we have drawn the phylogenetic tree using 25 kinds of insects for studying the relation in firefly family.

In this analysis, we used an adult male firefly, which was collected at Yamabe county, Nara prefecture, Japan (34°38′06.1″N, 136°02′19.4″E) and was stored in our laboratory at −80 °C. Genomic DNA was extracted by QIAGEN Blood & Cell DNA Min Kit and the sequencing was performed by Illumina Hiseq. The de novo sequence assembler Platanus was used for assembling process. Annotation was carried out by Bioedit v7.2.5 in reference to the mitogenome sequence of *L. cruciata* (AB849456). Annotated sequence was registered in GenBank with accession number LC306678.

The total length of assembled mitogenome was 16,719 bp, and has a typical gene content found in metazoan mitogenome, which contains 13 CDSs, 22 tRNA genes, two rRNA subunits, and an AT-rich region (Wolstenholme 1992). The composition of each base was calculated as A (42.50%), C (9.01%), G (14.16%), T (34.33%), and GC content was 23.17%. The start codon of COII and NDI was abnormal AAA and TTG, respectively. ATN codon was used in other 11 CDSs. In addition, an incomplete terminal codon namely single T was found in six CDSs (COII, COIII, ND3, ND5, ND4, and CytB). In case of other seven CDSs, TAA or TAG was used. The length of the AT-rich region was 2,100 bp, which is the third longest after the case of *A. leii* (2,239 bp) and *A. ficta* (2,240 bp) in firefly family (Jiao et al. 2015; Wang et al. 2017).

The neighbour-joining tree was constructed by MEGA6 with 1,000 bootstrap replicates using 25 kinds of insect including Coleoptera, Lepidoptera, Diptera, Hemiptera, Thysanura, Xiphosura, Lithobiomorpha, and Diplostraca (Figure 1). These species were as follows: *L. lateralis* (LC306678), *L. cruciata* (AB849456, LC306677), *L. substriata* (KP313820), *A. leii* (KF667531), *A. ficta* (KX758085), *A. wuhana* (KX758086), *Asymmetricata circumdata* (KX229747), *Pyrocoelia rufa* (AF452048), *Bicellonychia lividipennis* (KJ922151), *Crioceris duodecimpunctata* (AF467886), *Tribolium castaneum* (AJ312413), *Bombyx mori* (AF149768), *Bombyx mandarina* (NC003395), *Antheraea pernyi* (AY242996), *Anopheles quadrimaculatus* (NC000875), *Ceratitis capitata* (AJ242872), *Drosophila yakuba* (KF824901), *Chrysomya putoria* (AF352790), *Cochliomyia hominivorax* (AF260826), *Triatoma dimidiata* (AF301594), *Tricholepidion gertschi* (AY191994), *Limulus polyphemus* (AF216203), *Lithobius forficatus* (AF309492), and *Daphnia pulex* (KT003819). The bootstrap showed sufficient value at all nodes. It was found that *L. lateralis* was closer to the genus Aquatica than *L. cruciata* which is another famous aquatic firefly in Japan.

**Figure 1.**
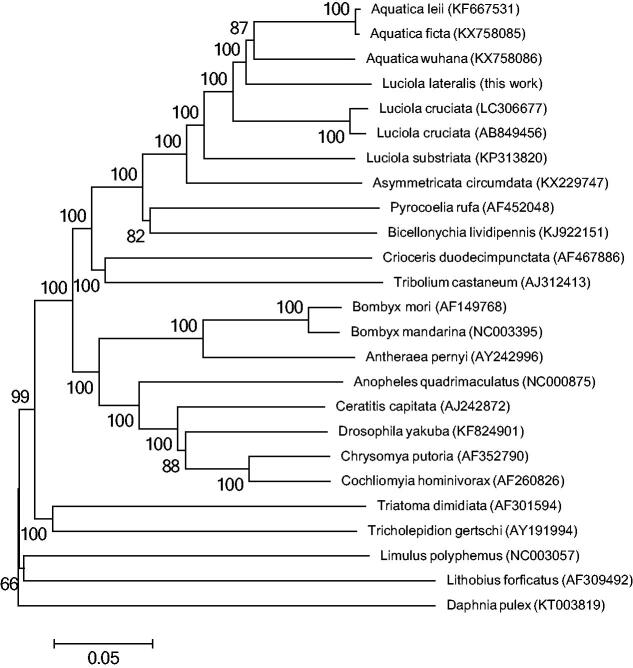
The phylogeny using complete mitochondrial genome sequence of 25 kinds of insect. The complete mitogenome of each species was obtained from GenBank and the phylogenetic tree was constructed by MEGA6 using neighbour-joining method with 1000 bootstrap replicates.
